# Comparing Monofractal and Multifractal Analysis of Corrosion Damage Evolution in Reinforcing Bars

**DOI:** 10.1371/journal.pone.0029956

**Published:** 2012-01-06

**Authors:** Yidong Xu, Chunxiang Qian, Lei Pan, Bingbing Wang, Chi Lou

**Affiliations:** 1 School of Materials Science and Engineering, Southeast University, Nanjing, Jiangsu, People's Republic of China; 2 Research Center of Green Building Materials and Waste Resources Reuse, Ningbo Institute of Technology of Zhejiang University, Ningbo, Zhejiang, People's Republic of China; University of Akron, United States of America

## Abstract

Based on fractal theory and damage mechanics, the aim of this paper is to describe the monofractal and multifractal characteristics of corrosion morphology and develop a new approach to characterize the nonuniform corrosion degree of reinforcing bars. The relationship between fractal parameters and tensile strength of reinforcing bars are discussed. The results showed that corrosion mass loss ratio of a bar cannot accurately reflect the damage degree of the bar. The corrosion morphology of reinforcing bars exhibits both monofractal and multifractal features. The fractal dimension and the tensile strength of corroded steel bars exhibit a power function relationship, while the width of multifractal spectrum and tensile strength of corroded steel bars exhibit a linear relationship. By comparison, using width of multifractal spectrum as multifractal damage variable not only reflects the distribution of corrosion damage in reinforcing bars, but also reveals the influence of nonuniform corrosion on the mechanical properties of reinforcing bars. The present research provides a new approach for the establishment of corrosion damage constitutive models of reinforcing bars.

## Introduction

The degradation of construction materials due to corrosion is of great concern for national economic development. The most important durability issue with concrete structure is deterioration due to reinforcing bar corrosion [1]. Considerable research work has been carried out on the deterioration of mechanical properties of corroded steel bars. While characterizing the corrosion degree of steel bars, most of the available studies adopt the index of corrosion mass loss ratio (denoted as S) [2]–[5]. Due to local attack penetration of chloride, the residual cross-section of corroded steel bars is no longer round and varies considerably along its circumference and its length. While corrosion mass loss ratio does not account for the nonuniform distribution of pitting corrosion. Given the same amount of mass loss in steel bars, localized corrosion seems to be a more dangerous damage type compared to uniform corrosion owing to more localized failure that brings about catastrophic fracture [6]. Therefore, how to quantitatively describe the corrosion characteristic and corresponding damage evolution are problems of current research.

From the damage mechanics point of view, the key in developing the relationship between the macro- and micro-material characteristics is the definition and selection of damage variables, which, at present, has no clear criteria to follow [7]. Since the concept of fractal was proposed by Mandelbrot [8], fractal geometry has been applied in many fields. Examples include the corrosion, roughness and fracture of metal materials [9]–[14]. These studies, however, are mainly focused on the monofractal characteristics of material damage and the fractal dimension (denoted as D) is considered as a suitable damage variable. For measure of Euclidean or fractal object, classical monofractal theory does not consider the singularity of local density. So the definition of density in classical physics loses its validity. Spontaneously the conception of singularity intensity is introduced and the distribution probability of singularity intensity is also considered, namely multifractal theory. Up to the present, there is little reference applying multifractal theory to damage evolution of corroded steel bars. Therefore, it is not clear whether the parameters of multifractal spectrum could be considered as a suitable damage variable to describe the corrosion evolution of reinforcing bars.

Based on fractal theory and damage mechanics, the aim of this paper is to describe the monofractal and multifractal characteristics of corrosion morphology and develop a new approach to characterize the nonuniform corrosion degree of reinforcing bars. By comparison, we here propose a multifractal damage variable, which can not only reflect the internal meso-scale corrosion damage but also facilitate the macro-scale analysis of damage mechanics. The relationship between the tensile strengths of the corroded steel bar and the multifractal damage variable is developed using laboratory test results for reinforcing bars of different corrosion levels. The results have important meaning for development of damage mechanics and solution of engineering problems.

## Materials and Methods

### Materials

**Figure 1:**
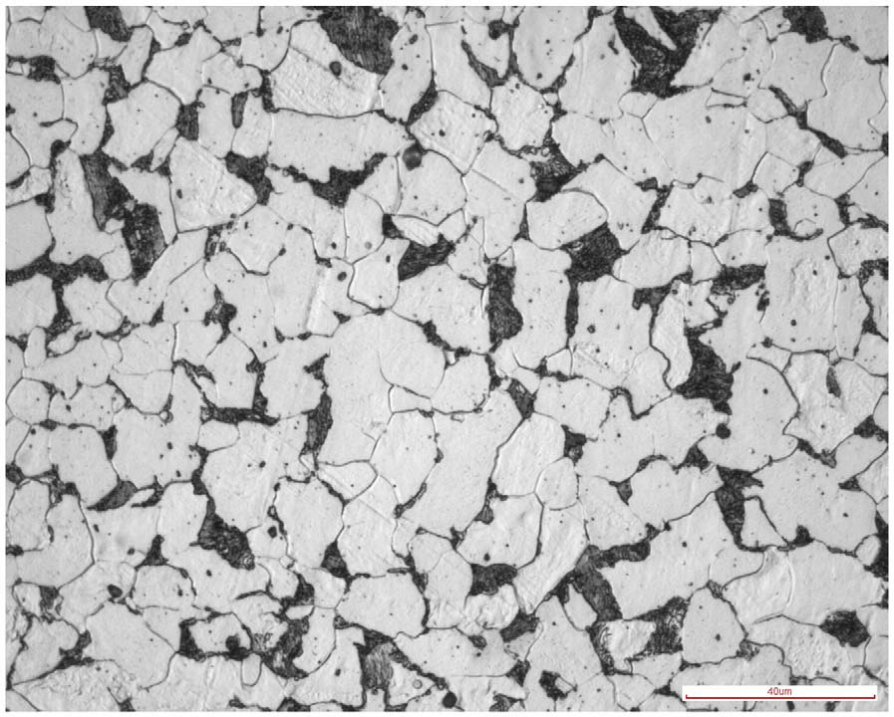
Metallographic structure of specimen. White part is ferrite and black part is pearlite.

Hot rolled plain steel bar (with nominal diameter of 12 mm) according to ISO Standards 6935-1 was used. The nominal carbon concentration is 0.18%. Figure 1 shows that the main metallographic structure consists of ferrite and pearlite. The salt solution was prepared by dissolving 5 parts by mass of sodium chloride (NaCl) into 95 parts of distilled water. The corresponding molar concentration is 0.9 M. The simulated concrete pore solution was made up of 0.6 M potassium hydroxide (KOH) + 0.2 M sodium hydroxide (NaOH) + 0.001 M calcium hydroxide (Ca(OH)_2_) [15]. The scale removal solution was prepared by mixing 3 parts by mass of hexamethylene tetramine into 97 parts diluted hydrochloric acid.

### Test Methods

**Figure 2:**
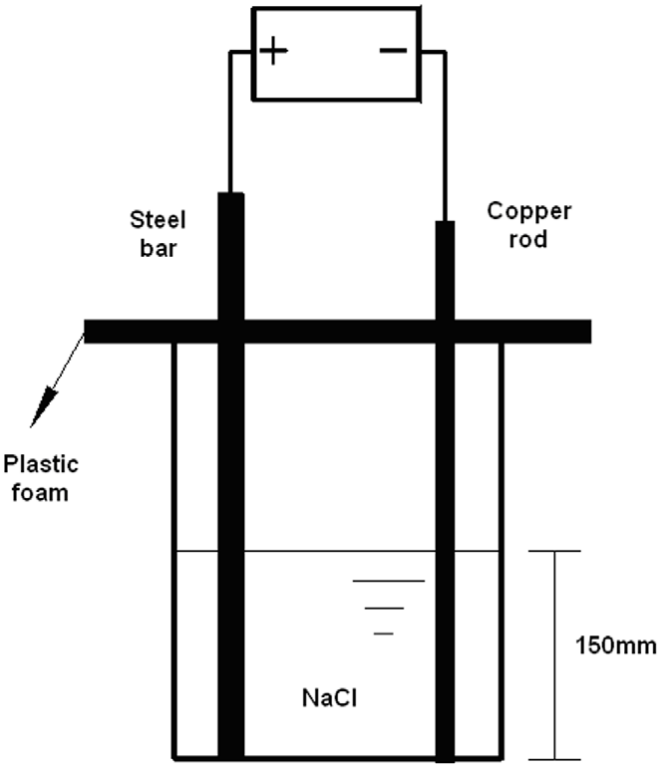
Schematic illustration of accelerated electrochemical corrosion test.

The specimen used for the accelerated electrochemical corrosion test was about 400 mm in length. Figure 2 shows a schematic representation of the experimental setup. The specimen was immersed into a jug of salt solution with 150 mm height. The detached part of the specimen was coated with insulation tape. The specimen was connected to the anode of a DC power source while the cathode of the DC power source was connected to a copper bar placed parallel to the specimen in the jug. The specimen was periodically washed by using a scale removal solution to remove corrosion products. After weighting, the corrosion mass loss ratio was calculated and the specimen continued to undergo the corrosion process.

**Figure 3:**
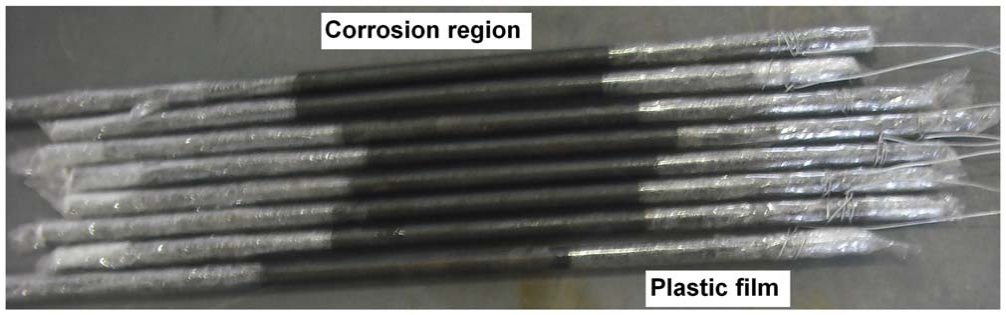
Specimens of alternative corrosion test. The two end parts of the specimen (each is about 125 mm) were coated with solid butter and plastic film and the middle part of the specimen (about 150 mm) was designed as the corrosion region.

The specimen used for the accelerated wet-dry cycle corrosion test was also about 400 mm in length and was cut from the same reinforcing bar as that used in the accelerated electrochemical corrosion test. The two end parts of the specimen (each is about 125 mm) were coated with anticorrosive grease and plastic film. The middle part of the specimen (about 150 mm) was designed as the corrosion region, as is shown in Figure 3. To determine the corrosion characteristics of reinforcing bar in chloride-free and chloride-contaminated simulated concrete solutions, eight specimens were subjected to wet-dry cycle corrosion test. Four specimens were regularly sprayed using the salt solution and the other four specimens were regularly sprayed using the mixture of salt solution and simulated concrete pore solution every 12 hours. When the test was completed, the specimens were washed using the scale removal solution to remove corrosion products. The corrosion mass loss ratio then was calculated.

The corrosion morphology images of the specimen were taken by using ME-61 stereomicroscope with magnification of 7X. To avoid the effect of the junction between the corrode and uncorroded parts, only the central region of 140 mm long in the corroded part was taken as the image sampling length. The corrosion morphology images were merged into one picture and then converted to binary images using ImageJ software. Tensile test was also performed for the specimen using standard strength test procedure according to ISO Standards 6892∶1998 to obtain the yield and ultimate strengths of the bar. In the tensile test an electro-hydraulic servo testing machine was used.

### Fractal Analysis Method

**Figure 4:**
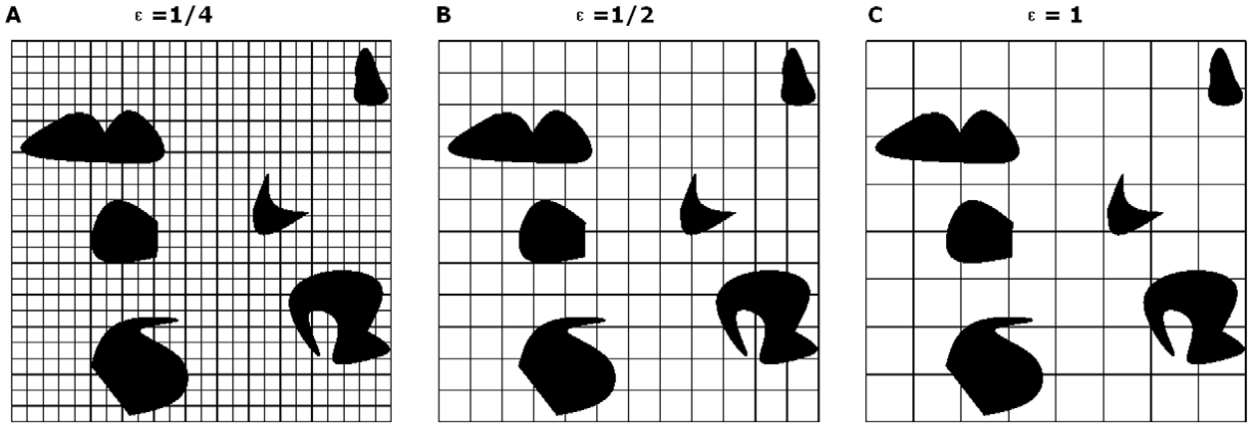
Schematic diagram of box counting method.

Fractal dimension is the most important parameter of monofractal theory. Many methods can be used to calculate fractal dimension, among which the box counting method is thought to be particularly suitable for the determination of corrosion morphology. In the box counting method it counts the number of square grids required to entirely cover an object surface, as is shown in Figure 4. By using different size grids, one can obtain a relation equation between the number of grids and the size of grids. Let ε be the side length of the grid, N(ε) be the number of grids required to cover the corroded area recorded in the image. According to monofractal theory, if an object is fractal, the number of grids and the size of the grids should have the following relationship, 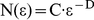
(1)where C and D are the constants. D is also called the fractal dimension. In order to explain how to determine these two constants, Eq.(1) is rewritten as follows,
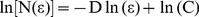
(2)By plotting the data set for ln[N(ε)] against ln(ε) and using the least-square fitting method, the fractal dimension parameter D can be easily obtained [16].

In terms of multifractal analysis, it is necessary to define a measure in the digital images which is closely associated with the local corrosion morphology. To calculate the multifractal spectrum, the following definition of measure was used [17]–[20]:
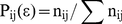
(3)where P_ij_(ε) is the gray value distribution probability in the box(i,j), n_ij_ is the gray value of the box(i,j) of size ε. P_ij_(ε) can be described as multifractal as
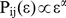
(4)

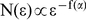
(5)where the exponent α depending upon the box (i, j) is the singularity of the subset of probabilities, N(ε) the number of boxes of size ε with the same gray value distribution probability, and f(α) the fractal dimension of the α subset. A quantity called partition function, χ_q_(ε), with an exponent τ(q) applied in statistical physics can be constructed as the following equation:
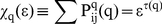
(6)where q is the moment order. τ(q) is evaluated by the slope of lnχ_q_(ε)∼ lnε curve. A generalized multifractal spectrum function, f(α), can then be calculated through Legendre transform:
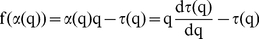
(7)Multifractal measures are primarily characterized by their spectrum. The plot of f(α)∼ α is called multifractal spectrum, which is generally a hook-shaped curve. The width of the multifractal spectrum is Δα and the difference of the fractal dimensions of the maximum probability (α = α_min_) and the minimum one (α = α_max_) is Δf (Δf = f(α_min_)-f(α_max_)).

The generalized dimension, D(q), addresses how mass varies with ε in an image which are calculated from the mass exponent function:
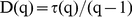
(8)For a non- or monofractal the plot of D(q) versus q tends to be horizontal or non-increasing, but for a multifractal, it is generally sigmoidal and decreasing.

In general, the related fractal parameters (D and Δα) increase with the increase of the complexity of the corrosion morphology, which can indirectly characterize the corrosion damage of reinforcing bars. For calculating the related fractal parameters, the free plugin FracLac of ImageJ is used.

## Results and Discussion

### Fractal Evolution of Corroded Reinforcing Bars

**Figure 5:**
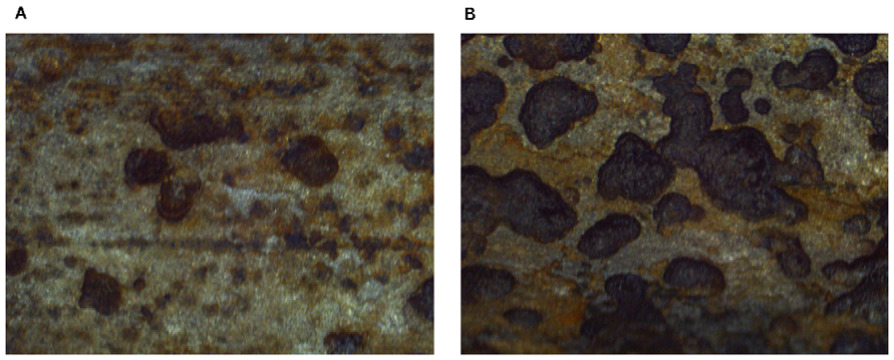
Local corrosion morphology of corroded reinforcement. (A) Low corrosion damage degree with slight localize attack, S = 2.4%, (B) High corrosion damage degree with severe localize attack, S = 9.6%.

Figure 5 shows the typical images of corrosion morphology for the same specimen at different corrosion levels obtained in accelerated electrochemical tests. These corrosion morphologies have generally irregular shapes and orientations. Some of the local corrosion morphologies are connected together, while others are isolated. The complexity of the corrosion morphology increase generally with the increase of corrosion mass loss ratio, which indicating the damage degree of steel bars increase with increasing disorder. The calculation of fractal dimension and multifractal spectrum has been performed and the related parameters are summarized in Table 1.

**Table 1:** Fractal parameters for describing the corrosion distribution in reinforcing bars.

S (%)	D	Δα	Δf
2.4	1.68	1.01	0.63
4.3	1.71	1.17	0.42
6.4	1.72	1.40	0.90
9.6	1.75	1.64	0.55
12.3	1.76	1.69	0.57

**Figure 6:**
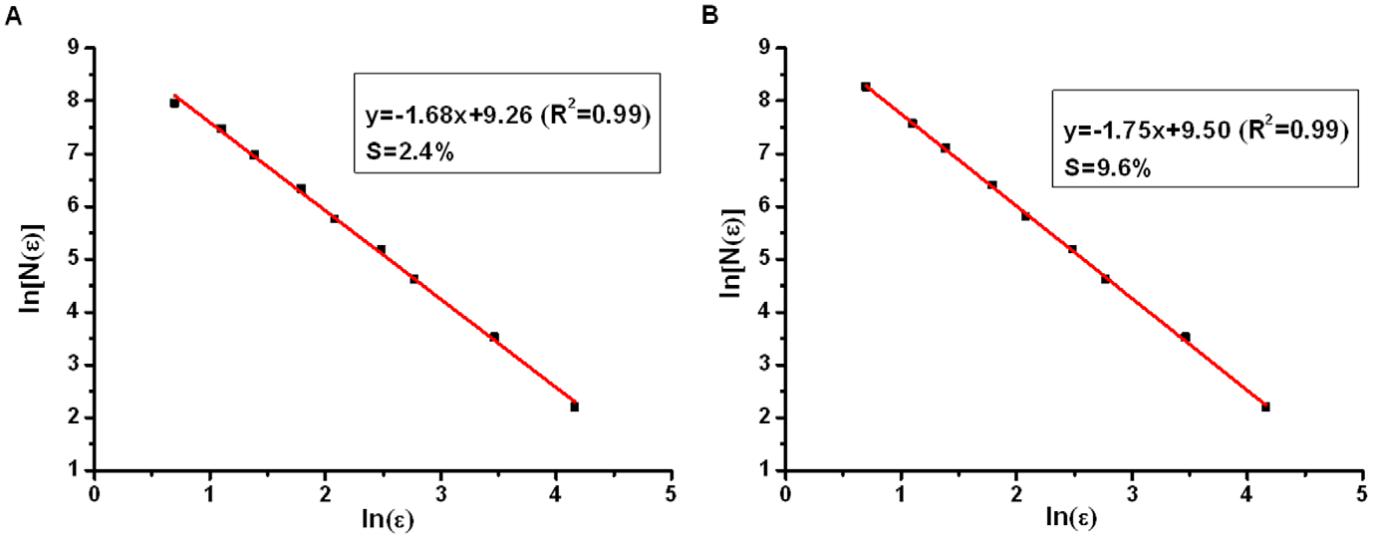
Fractal dimension of corrosion morphology.

As for monofractal analysis, fractal dimension of corrosion morphologies with different corrosion levels is calculated by box counting method. As is shown in Figure 6, the correlation coefficient of linear regression model is 0.99, which indicates that the distribution of corrosion morphology exhibits a statistical fractal feature. The fractal dimensions of corrosion morphology are between 1 and 2, which increase generally with increase of corrosion mass loss ratio.

**Figure 7:**
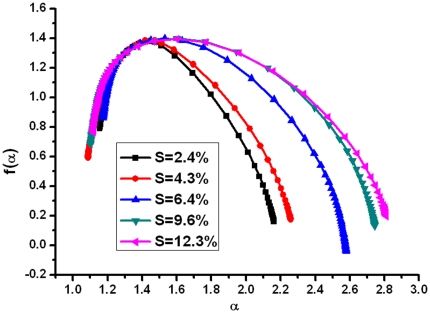
Multifractal spectra of corrosion morphology.

**Figure 8:**
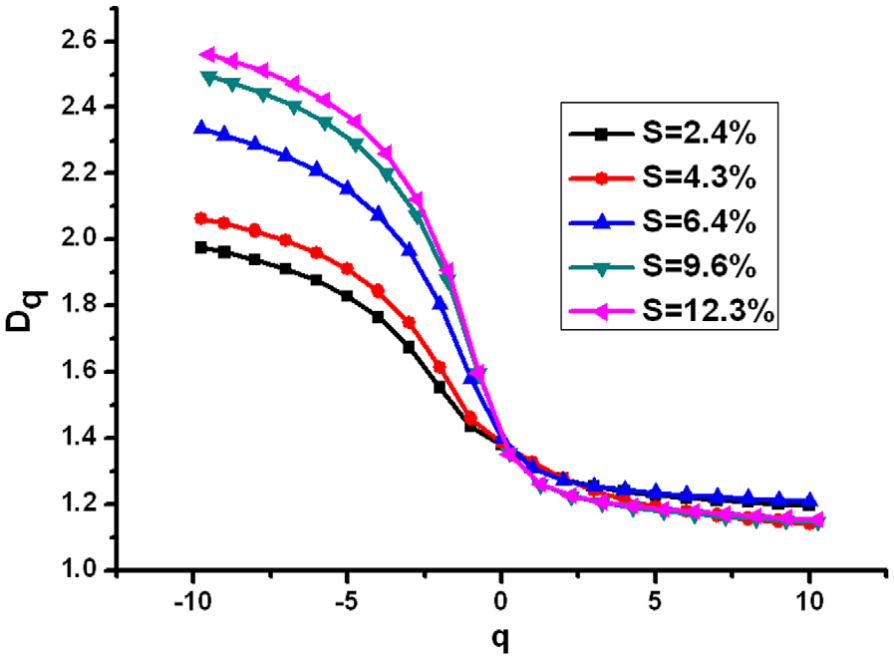
Generalized dimensions spectra D(q) at −10 to 10 ranges of q evaluated at 1.0 increments.

As for multifractal analysis, Figure 7 plots the multifractal spectrums of corrosion morphologies with different corrosion levels. All the multifractal spectrums are humped and mainly like hooks to the left, which indicates the existence of multifractal in corrosion morphology. All the Δf values are greater than 0, which indicating the distribution of corrosion depends on the maximum subset of probability. Generally, the higher the value of Δα is, the more inhomogeneous distribution patterns will be. With the increase of the corrosion mass loss ratio, there is an increase in the width of the multifractal spectrum (Δα) [17], [20]. The generalized dimensions spectra D(q) are shown in Figure 8. In all cases, D(q) is a decreasing function. This further indicates that the distribution of corrosion morphology exhibits multifractal feature [19]. The results demonstrate that the related fractal parameters, such as D and Δα, are index of its morphometric variability and complexity. The damage evolution process in the reinforcement corrosion can be described using the fractal geometry theory.

### Relationship between Fractal Damage Variable and Tensile Strength of Corroded Reinforcing Bars

**Figure 9:**
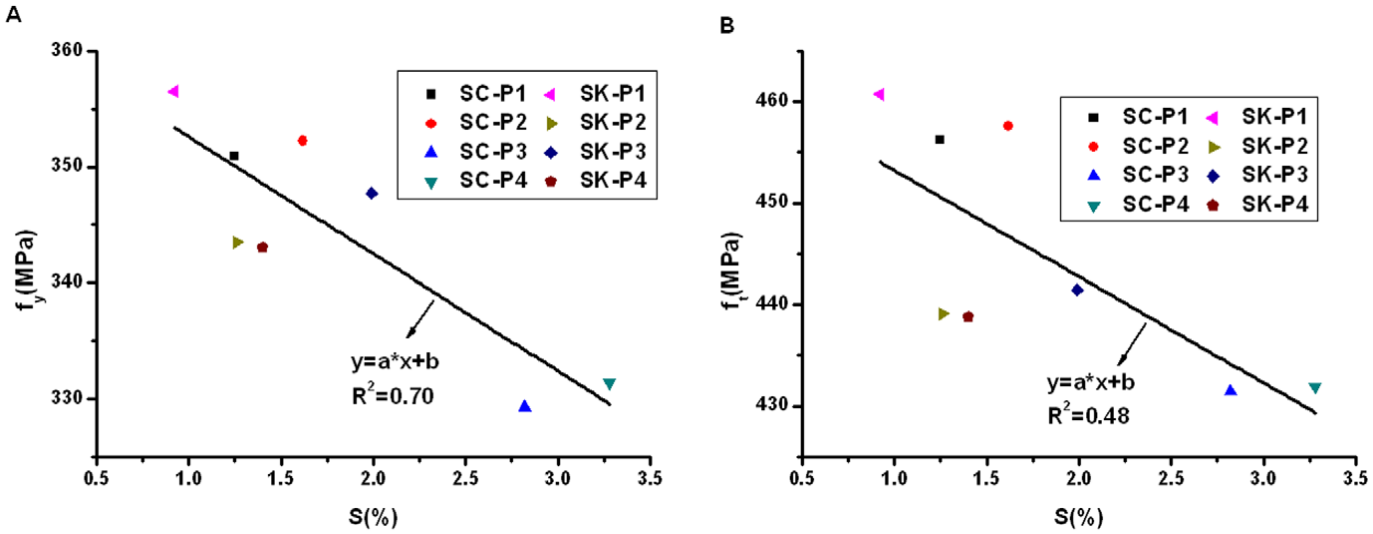
Relationship between corrosion mass loss ratio and tensile strengths.

**Figure 10:**
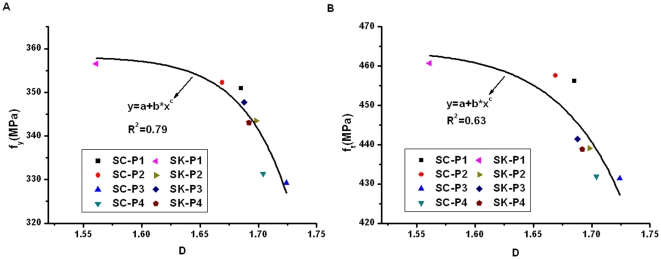
Relationship between fractal dimension and tensile strengths.

**Figure 11:**
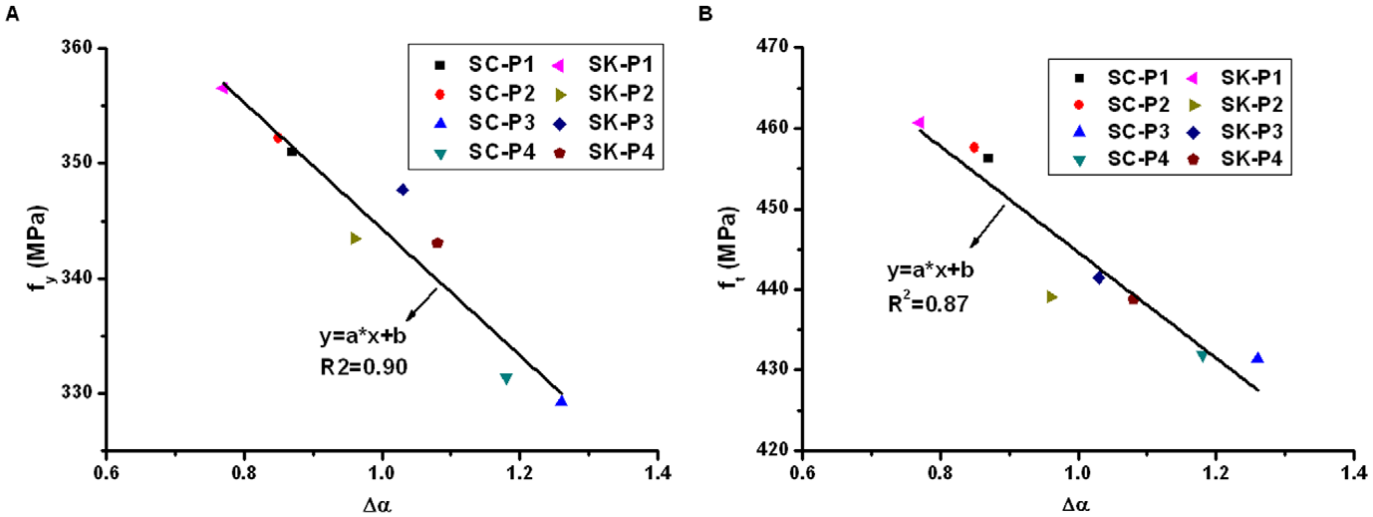
Relationship between width of multifractal spectrum and tensile strengths.

In order to demonstrate whether the related fractal parameter can also reflect the corrosion damage in reinforcing bars subjected to different aggressive environments, we calculate the fractal dimension and multifractal spectrums for the eight bars tested using the accelerated wet-dry cycle corrosion method. Four specimens, with code named SC-P1∼SC-P4, were regularly sprayed using salt solution. The other four specimens, with code named SK-P1∼SK-P4, were regularly sprayed using the mixture of salt solution and simulated concrete pore solution. The results, together with the corrosion mass loss ratio obtained from the corrosion test, yield and ultimate strengths obtained from the tensile test, are plotted graphically in Figures 9, 10, and 11.

As is shown by Figure 9, both the yield strength and ultimate strength generally decrease with the increase of corrosion mass loss ratio, but the linear relationship between them seems not very good. Under condition of similar corrosion mass loss ratio, as is shown by SC-P1 and SK-P2 specimens, it does show that the two bars, which have similar corrosion mass loss ratio, have very different tensile strengths. While for SC-P1 and SC-P2 specimens, it does show that the two bars, which have different corrosion mass loss ratio, also have similar tensile strengths. As we know, the index of corrosion mass loss ratio can be applied to the corroded bars of uniform corrosion. However, the corrosion of reinforcing bars is often caused due to chloride attack, which is a “localized” pitting corrosion. Therefore, corrosion mass loss ratio of a bar cannot accurately reflect the damage degree of the bar.

Figure 10 plots the variation of the tensile strength with the fractal dimension. The experimental data are fitted with a power function, which seems reasonably good. Both the yield and ultimate strengths initially decrease slowly with the increase of fractal dimension. Only after the fractal dimension exceeds about 1.69, the tensile strengths of the corroded steel bar begin to decrease remarkably. The results show that, in the early stage of corrosion, the damage is generally manifested as the growth of the irregular distribution of corrosion morphology, which indicates that the fractal dimension of corrosion morphology increases rather quickly. However, after a certain level of corrosion is reached, the corrosion damage becomes mainly in the increase of corrosion pitting depth, while the development of surface corrosion morphology becomes slow and not obvious. Owing to the classical monofractal theory does not consider the singularity of local density, leading the less change in fractal dimension and higher change in tensile strength. Comparatively, monofractal can only reflect the degree of reinforcement damage and its evolution to a certain extent. But fractal dimension is still not an ideal damage variable.

It can be seen from Figure 11 showing the relationship between the width of multifractal spectrum (Δα) and the corrosion mass loss ratio that, in general, the experimental data are fitted with a linear regression model. As mentioned above, classical monofractal theory does not consider the singularity of local density. While a multifractal object is more complex in the sense that it is always invariant by translation, although the dilatation factor needed to be able to distinguish the detail from the whole object depends on the detail being observed. Multifractal could be seen as an extension of monofractal. It solves the problem that fractal dimension cannot adequately characterize the structure and property of a fractal object [21], [22]. In contrast, it is more consistent when the width of multifractal spectrum (Δα) of corrosion morphology is used to represent the corrosion damage. Both the yield strength and ultimate strength generally decrease with the width of multifractal spectrum (Δα), and the linear relationship between them seems very good. This indicates that the damage evolution process in the reinforcement corrosion can be described using the multifractal theory. The results have demonstrated that using the width of multifractal spectrum (Δα) as the damage variable can not only reflect the distribution of corrosion damage in reinforcing bars, but also reveal the influence of nonuniform corrosion on the mechanical properties of the corroded reinforcement. The analysis may become the basis for the development of corrosion damage constitutive models of reinforcing bars.
